# A dual-sensitized luminescent europium(iii) complex as a photoluminescent probe for selectively detecting Fe^3+^†

**DOI:** 10.1039/d0ra03821k

**Published:** 2020-06-25

**Authors:** Yafeng Zhao, Yanhong Xu, Bing Xu, Peipei Cen, Weiming Song, Lijuan Duan, Xiangyu Liu

**Affiliations:** College of Agriculture, College of Chemistry and Chemical Engineering, Ningxia University Yinchuan 750021 China xiangyuliu432@126.com songwm@nxu.edu.cn; College of Public Health and Management, Ningxia Medical University Yinchuan 750021 China 13895400691@163.com; School of Chemistry and Chemical Engineering, Xi'an University of Architecture & Technology Xi'an 710055 China; State Key Laboratory of Coordination Chemistry, Nanjing University Nanjing 210023 China

## Abstract

A new luminescent Eu^III^ complex, namely [Eu_2_(BTFA)_4_(OMe)_2_(dpq)_2_] (1), in which BTFA = 3-benzoyl-1,1,1-trifluoroacetone and dpq = dipyrido [3,2-*d*:2′,3′-*f*] quinoxaline, has been designed and synthesized by employing two different ligands as sensitizers. Crystal structure analysis reveals that complex 1 is composed of dinuclear Eu^III^ units crystallized in the monoclinic *P*1̄ space group. Notably, 1 exhibits high thermal stability up to 270 °C and excellent water stability. The photoluminescence property of the complex is investigated. Further studies show 1 can recognize Fe^3+^ ions with high selectivity from mixed metal ions in aqueous solution through the luminescence quenching phenomenon. Furthermore, the recyclability and stability of 1 after sensing experiments are observed to be adequate. By virtue of the superior stability, detection efficiency, applicability and reusability, the as-prepared Eu^III^ complex can be a promising fluorescent material for practical sensing.

## Abstract 2

A stable dual-sensitized Eu(iii)-complex constructed with two different ligands can be used as a sensing platform for Fe^3+^ detection.

## Graphical abstract

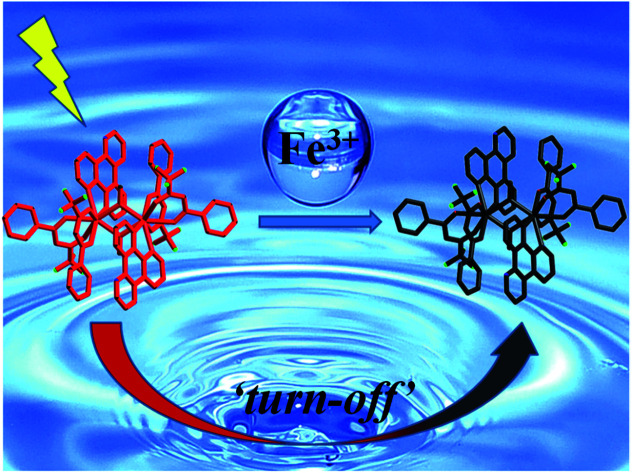

## Introduction

As one of the most essential trace elements in the human body, iron ions play essential roles in many biochemical processes including cellular metabolism, oxygen uptake, DNA/RNA synthesis and enzyme catalysis.^1–3^ The permissible limit of iron in drinking water given by the World Health Organization (WHO) is 0.3 mg mL^−1^.^4^ Both a deficiency and excess of Fe^3+^ from the normal permissible limit will break cellular homeostasis and cause serious biological disorders like microcytic hypochromic anemia and Alzheimer's disease.^5–7^ Thus it is of great urgency to obtain effective methods for probing the trace of Fe^3+^ ions. To date, existing techniques have been put forward for Fe^3+^ detection including liquid chromatography, inductively coupled plasma atomic mass spectrometry (ICP-MS) and atomic absorption spectrophotometry (AAS).^8–10^ While all the above-mentioned ways offer advantages, none is ideal due to certain features such as lack of portability, being time-consuming, high cost and requiring expertise.

Compared to traditional instrumental and chemical methods, chemsensors have received wide attention due to their short response time, excellent sensitivity, simplicity and low cost. Organic–inorganic hybrid Ln^III^ complexes have been attracted as promising luminescent probes for sensing applications due to their unique spectroscopic and chemical properties such as long luminescence lifetime, large Stokes shift, sharp emission bands, as well as good mechanical, thermal, and chemical stability.^11–13^ However the Laporte-forbidden 4f–4f transition prevents direct excitation of lanthanide luminescence, Ln^III^ ions always require sensitization by suitable organic chromophores. Furthermore, for practical applications, Ln^III^ ion must be incorporated into highly stable coordinated complexes. The efficiency of ligand-to-metal energy transfer, which requires compatibility between the energy levels of the ligand excited states and accepting levels of Ln^III^ ions, is crucial in the design of high performance luminescent molecular devices. The β-diketone ligand is one of the important “antennas”,^14^ from which the energy can be effectively transferred to Ln^III^ ions for high harvest emissions and has the following advantages. The β-diketone ligand has strong absorption within a large wavelength range for its π–π* transition, consequently, being targeted for its ability to sensitize the luminescence of the Ln^III^ ions. Meanwhile, it has ability to form stable and strong adducts with Ln^III^ ions, which can have practical usage.^15,16^ Therefore, 3-benzoyl-1,1,1-trifluoroacetone (BTFA) is selected as the main ligand. Besides, the advantages of the mixed ligands synthetic strategy inspired us to introduce other versatile bridging linkers into the reaction system. Herein, dipyrido [3,2-*d*:2′,3′-*f*] quinoxaline (dpq) is selected as an auxiliary ligand. On the one hand, it has four potential coordination domains to coordinate with the Ln ions. On the other hand, it is born with conjunctive aromatic rings, which not only increases the rigidity of the structures but also provides a powerful absorbing sensitizer.^17–20^

**Scheme 1:**
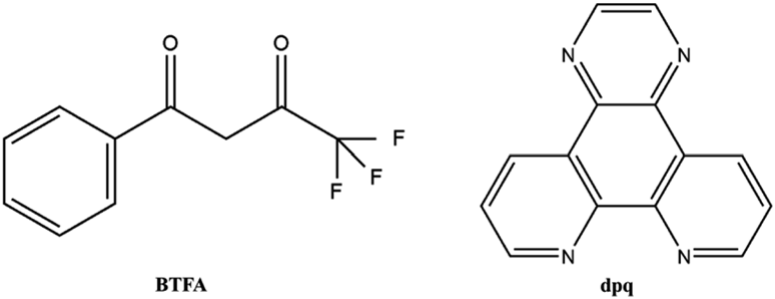
Graphical representation of BTFA and bpq ligands.

Using this strategy, we prepare a novel luminescent Eu^III^ complex [Eu_2_(BTFA)_4_(OMe)_2_(dpq)_2_] (1) based on the BTFA ligand and dpq coligand (Scheme 1). Single-crystal structure analysis demonstrates that 1 is a bimetallic construction in which the Eu^III^ ion is best described as an eight-coordinated square antiprism geometry (SAPR-8, *D*_4d_, 0.626) with moderate distortions from the ideal geometry.^21^ The complex integrates the advantages of two functional ligands and the photoelectric units of Eu^III^, exhibiting favorable stability and superior fluorescence properties. Moreover, 1 is indicative of a highly selective luminescence quenching action for Fe^3+^ cation in aqueous media. The strategy here for preparing stable Eu^III^ complex is very simple and feasible for large-scale synthesis, which would open a new way towards the functional applications in chemical sensor.

## Experimental

### Materials and physical measurements

All chemicals and solvents were of reagent grade, obtained from commercial sources without further purification. Fourier transform infrared (FT-IR) spectra were recorded in the range 400–4000 cm^−1^ using KBr pellets on an EQUINOX55 FT/IR spectrophotometer. Elemental analysis (C, H, N) was performed with a PerkinElmer 2400 CHN elemental analyzer. The phase purity of the bulk or polycrystalline samples was confirmed by powder X-ray diffraction (PXRD) measurements executed on a Rigaku RU200 diffractometer at 60 kV, 300 mA, and Cu Kα radiation (*λ* = 1.5406 Å), with a scan speed of 5° min^−1^ and a step size of 0.02° in 2*θ*. Thermal gravimetric analyses (TGA) were performed by using a NETZSCH STA 449F3 instrument at a heating rate of 10 °C min^−1^ from 30 to 800 °C and under a N_2_ stream using an empty Al_2_O_3_ crucible as the standard. The fluorescence spectra were obtained using a Hitachi F-7000 fluorescence spectrophotometer at room temperature. UV-Vis spectroscopic studies were collected on shimadzu WFH-203B spectrophotometer.

### Synthesis of [Eu_2_(BTFA)_4_(OMe)_2_(dpq)_2_] (1)

A mixture of Et_3_N (0.014 mL, 0.2 mmol) and BTFA (0.0648 g, 0.3 mmol) in methanol (20 mL) was kept stirring for an hour, to which EuCl_3_·6H_2_O (0.1 mmol) and dpq (0.0464 g, 0.2 mmol) were introduced. After stirring for 4 h, the resulting mixture was immediately filtrated, and the filtrate was left to stand at room temperature for slow evaporation. Colorless crystals were generated by slow evaporation of the filtrate after several days, obtain a 55.3% yield of 1 (based on Eu^III^). Elemental analysis: (%) calcd for C_70_H_46_Eu_2_F_12_N_8_O_10_ (1691.07): C, 49.72; H, 2.74; N, 6.63. Found: C, 49.70; H, 2.72; N, 6.70. Main IR (KBr, cm^−1^): 3424 (w), 1638 (m), 1611 (s), 1576 (s), 1530 (m), 1403 (w), 1321 (m), 1294 (s), 1184 (s), 1141 (s), 944 (w), 767 (s), 701 (m), 580 (m).

### Crystallographic data collection and refinement

The X-ray experiments were implemented on a Bruker SMART APEX-CCD-based diffractometer (Mo Kα radiation, *λ* = 0.71073 Å) at low temperature. Using Olex2,^22^ the structure of 1 is solved with the ShelXT^23^ structure solution program by using Intrinsic Phasing, and refined with the ShelXL^24^ refinement package by using Least Squares Minimisation. All the non-hydrogen atoms are refined anisotropically. All the hydrogen atoms of complex 1 are located from difference maps by the program Olex2. Crystallographic data and refinement parameters are listed in Table 1, while selected interatomic distances and angles for complex 1 are given in Table S1.†

**Table 1:** Crystal data and structure refinement details for 1

Complex	1
Empirical formula	C_70_H_46_Eu_2_F_12_N_8_O_10_
Formula weight	1691.07
Temperature	100(10) K
Crystal system	Triclinic
Space group	*P*1̄
*a* (Å)	10.2450(12)
*b* (Å)	12.4101(10)
*c* (Å)	14.5367(13)
*α* (°)	112.234(8)
*β* (°)	106.909(10)
*γ* (°)	94.287(8)
*V* (Å^3^)	1600.8(3)
*Z*	1
*D* (g cm^−3^)	1.754
Mu (mm^−1^)	2.045
*F*(0 0 0)	836.0
Unique reflections	7443
Observed reflections	12 478
*R*_int_	0.0635
Final *R* indices [*I* > 2*σ*(*I*)]	*R*_1_ = 0.0714 w*R*_2_ = 0.1437
*R* indices (all data)	*R*_1_ = 0.0979 w*R*_2_ = 0.1600
Goodness-of-fit on *F*^2^	1.011

### Fluorescence measurements

The powder sample (3 mg) of 1 was respectively immersed in 0.01 mol L^−1^ of various M(NO_3_)_*x*_ (M = Co^2+^, NH_4_^+^, Mg^2+^, Cr^3+^, Pb^2+^, Ag^+^, Na^+^, Cd^2+^, Zn^2+^, Cu^2+^, Li^+^, Ca^2+^, K^+^, Mn^2+^, Ni^2+^ and Fe^3+^) aqueous solutions (5 mL) for 2 days at room temperature. Then, all the mixtures should be subjected to ultrasonication for 30 min to produce a stable suspension. Subsequently, the fluorescence spectrum of the MOF suspension was recorded. For selective experiments, 3 mg of the powder sample of 1 was submerged into kinds of aqueous solutions containing Fe^3+^ ions and other metal ions (5 mL, 0.01 mol L^−1^ of Co^2+^, NH_4_^+^, Mg^2+^, Cr^3+^, Pb^2+^, Ag^+^, Na^+^, Cd^2+^, Zn^2+^, Cu^2+^, Li^+^, Ca^2+^, K^+^, Mn^2+^ and Ni^2+^) for 2 days. Also, we used an identical method to treat the mixed solutions before the fluorescence test. For the detection of Fe^3+^ ions, 3 mg of the powder sample of 1 was respectively submerged into aqueous solutions (5 mL) with various concentrations of Fe^3+^ ions for 2 days; then, its fluorescence intensity was measured after obtaining a uniform MOF suspension.

### Recyclable luminescence experiments

The recyclability of 1 towards sensing Fe^3+^ was also investigated. After the first quenching experiment, the powder of 1 was centrifuged and washed several times with deionized water. The recovered solid was collected and then used in the successive quenching experiments.

## Results and discussion

### Crystal structure of 1

**Fig. 1:**
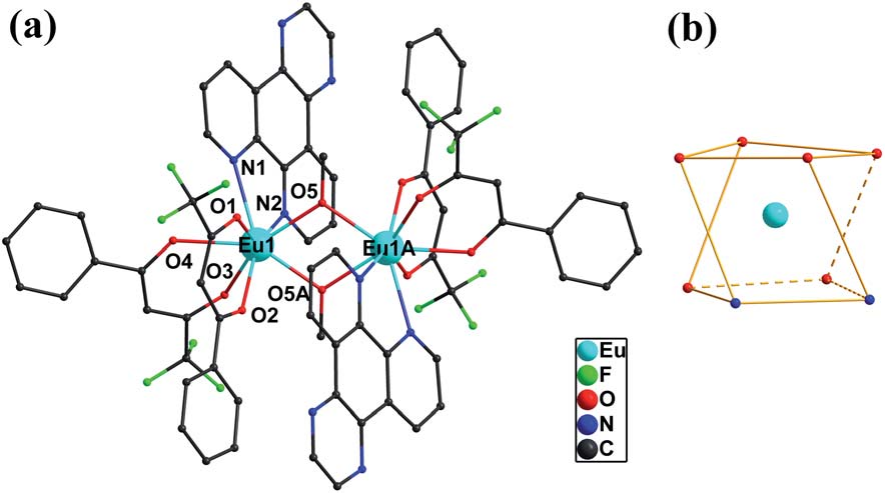
Molecular structure of 1 (a) and coordination geometry around the Eu ion for 1. (b) All hydrogen atoms are omitted for clarity.

Single-crystal X-ray structural analysis reveals that complex 1 is centrosymmetric and crystallizes in the monoclinic *P*1̄ space group (Table 1). As shown in Fig. 1a, 1 features dinuclear units, consisting of two Eu^III^ ions, four BTFA ligands, two dpq motif and two methanol molecules. The coordination environments of two Eu^III^ ions are equivalent to each other. Each Eu^III^ ion has a [EuN_2_O_6_] coordination sphere with two nitrogen atoms (N1 and N2) from one dpq motif ligand, four oxygen atoms (O1, O2, O3 and O4) from two BTFA ligands moiety, and two oxygen atoms (O5 and O5A) from two methanol molecules. Two Eu centers are dual-bridged by oxygen atoms from two methanol molecules, forming a dinuclear unit with Eu1⋯Eu1A distances of 3.770 Å and Eu1–O2–Eu1A angles of 109.19. In the crystal packing (Fig. S1†), the dinuclear Eu_2_ unit is further strengthened through π⋯π (3.5301(3) Å) interactions. The shortest intermolecular Eu⋯Eu distances are 9.838 Å, suggesting spatial isolation of the dinuclear motifs.^25^ To ascertain the precise geometries around the metallic centers and the degree of the distortion from the ideal model for Eu complex, the geometric spheres of Eu^III^ cations are calculated by using the SHAPE 2.1 program^26^ based on the structural parameters; the typical geometric polyhedrons are depicted in Fig. 1b. In principle, the data extracted from the package tend to zero, responding to the optimal geometry, whereas a greater value presents a major deviation from the optimal polyhedron.^27^ As shown in Table S2,† the calculation indicates that Eu^III^ centers are best described as eight-coordinated square antiprism geometry (CshMs parameters: 0.626).

### PXRD and thermal analysis

The phase purity of the synthesized complex 1 is demonstrated by PXRD. As displayed in Fig. S2,† all experimental peaks are in agreement with those in the simulated profile derived from single-crystal diffraction data. The result demonstrates the high phase purity of 1. Aiming to investigate the thermal stabilities of 1, the thermal gravimetric (TG) analyses of 1 is performed (Fig. S3†). According to the TGA curve, 1 almost do not lose any obvious weight until 270 °C, suggesting no guest molecules in the lattice, as confirmed by the single crystal X-ray diffraction analysis. Subsequently, the framework begins to decompose, implying a certain thermostability existing in the complex.

### Water stability

Most MOFs have shown limitations in practical applications due to low stability, especially when exposed to water. To further explore the water stability of MOFs is significant to practical applications. The stability experiments of 1 are executed *via* immersing crystals of 1 in water for three days at room temperature. The obtained PXRD patterns remain intact, indicating that no phase transition or framework collapse occurred during the treatments (Fig. S4†). After immersing in water for 3 days, no signals observed in the NMR spectrum showed that there is a poor solubility in water. Moreover, CHN analysis (C, 49.74%; H, 2.75%; N, 6.68%) after prolonged exposure to water has been carried out, further confirming that the MeO-bridges could not be exchangeable with the OH bridges. As shown in Fig. S5,† NMR experiment in manual operation reveals that the MeO groups still exist in the complex, confirming that the complex can be dissolved in MeOH and is thus soluble. The results obtained reveal that 1 possesses satisfactory water stability.

### Fluorescence properties

**Fig. 2:**
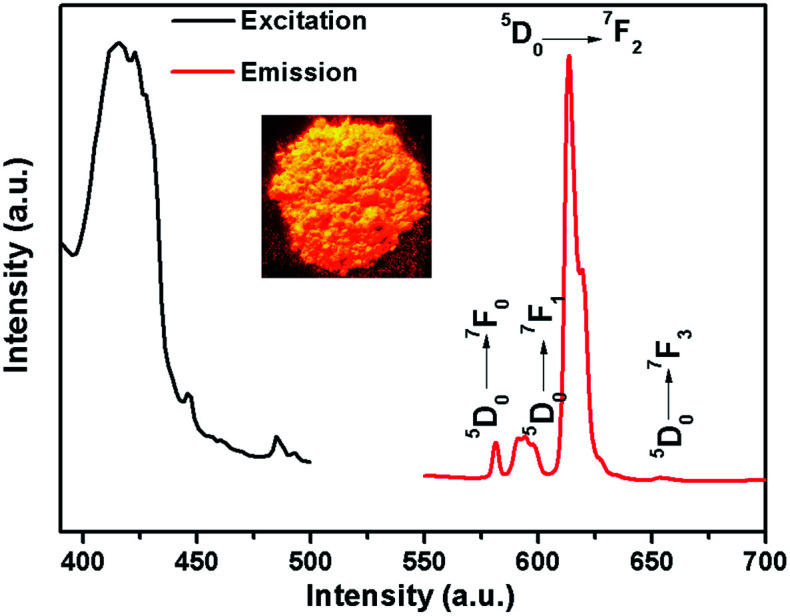
The excitation (black line) and emission spectra (red line: *λ*_ex_ = 420 nm) of 1.

**Fig. 3:**
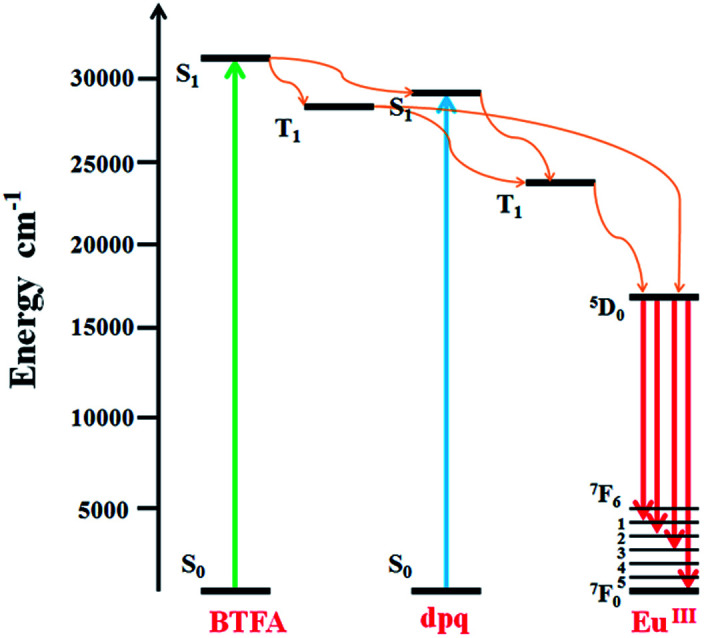
Schematic energy-level diagram showing the intramolecular ET between the ligands and Eu^III^; *S*_1_ = first excited singlet state; *T*_1_ = first excited triplet state.

Because of partial filling of the 4f shells of Ln^3+^, Ln-complexes have shown good luminescence properties including sharp feature emissions, large Stokes shifts, relatively long luminescence lifetimes, and bright luminescent colors, especially for the Eu-complexes.^28^ In order to determine the luminescent properties of the system, the solid-state excitation and emission spectra of 1 was studied. The incorporation of the two ligands shifts the excitation window towards a favorable longer wavelength region. As shown in Fig. 2, the solid fluorescence spectra of 1 showed characteristic emission peaks of Eu^III^ under excitation at 420 nm. The peaks at 581, 593, 613 and 653 nm could be assigned to ^5^D_0_ → ^7^F_0_, ^5^D_0_ → ^7^F_1_, ^5^D_0_ → ^7^F_2_, and ^5^D_0_ → ^7^F_3_ transitions, respectively. In dual-sensitized [Eu_2_(BTFA)_4_(OMe)_2_(dpq)_2_] (1), the energy transfer from the dual antenna to the Eu^III^ is more facile because of efficient intersystem crossing (ISC) and optimal ET from the ligand triplet states to the emissive excited state (^5^D_0_) of Eu^III^. This results in a characteristic brilliant red luminescence due to ^5^D_0_ → ^7^F_*J*_ (*J* = 0–3) transitions dominated by electric dipole (ED)-induced hypersensitive ^5^D_0_ → ^7^F_2_ (613 nm) transitions.^29,30^ The ^5^D_0_ → ^7^F_1_ transition for Eu-MOF at 593 nm was attributed to the magnetic dipole transition, and the intensity was independent of the local environment of the Eu^3+^. The most powerful emission at 613 nm was attributed to the electric dipole induced ^5^D_0_ → ^7^F_2_ transition, which was hypersensitive to the coordination environment of Eu^3+^.^31^ The luminescence intensity at 613 nm (^5^D_0_ → ^7^F_2_ transitions) is stronger than at 593 nm (^5^D_0_ → ^7^F_1_ transitions), which indicates that Eu^3+^ ions take up non-inversion center. For an effective ET process in Eu^III^, the ^3^T energy level of the ligands should be higher than the ^5^D_0_ level of Eu^III^ (17 200 cm^−1^) and ideally the hydration number, *q*, should be zero. An optimal L → Ln^III^ ET process depends on ligands that can efficiently transfer energy to the triplet states through ISC. Moreover, the energy gap (DE) between the ligand ^3^T states (*e.g.*, ^3^n–π*, ^3^π–π*) and the emissive excited states of Eu^III^ (^5^D_0_) ideally should be ≥2500 cm^−1^.^32^ The singlet (*S*_1_) and triplet (*T*_1_) energy levels of the BTFA (31 996 and 28 488 cm^−1^) and dpq (29 400 and 23 500 cm^−1^) are provided to semi-quantitively evaluate the ET processes of the Eu^IIII^ complex.^20^ The schematic ET processes for the complexes are shown in Fig. 3. On account of the superior water stability, thermal stability and luminescence properties, 1 can be identified as a feasible complex that exhibits potential application performance in fluorescence sensing.

### Detection of metal ions

**Fig. 4:**
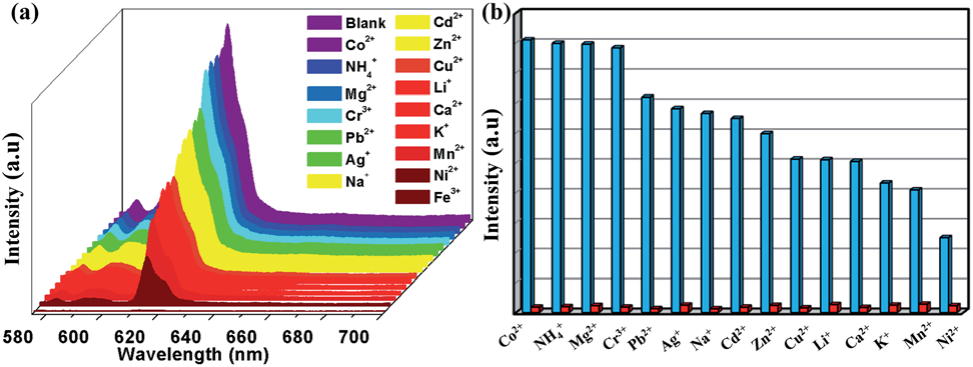
(a) Luminescent intensity (613 nm) of 1 treated with 0.01 M of various different metal ions in water. (b) Luminescence intensity at (613 nm) of 1 dispersed in water with the addition of different ions (0.01 M) (Co^2+^, NH_4_^+^, Mg^2+^, Cr^3+^, Pb^2+^, Ag^+^, Na^+^, Cd^2+^, Zn^2+^, Cu^2+^, Li^+^, Ca^2+^, K^+^, Mn^2+^, Ni^2+^) and Fe^3+^-incorporated systems (0.01 M).

Considering the desired excellent water stability and fluorescence performance of 1, we investigate the sensing capability of this Eu-complex towards various metal ions in water. The powder samples of 1 are immersed in 0.01 mol L^−1^ of various M(NO_3_)_*x*_ aqueous solutions, including Co^2+^, NH_4_^+^, Mg^2+^, Cr^3+^, Pb^2+^, Ag^+^, Na^+^, Cd^2+^, Zn^2+^, Cu^2+^, Li^+^, Ca^2+^, K^+^, Mn^2+^, Ni^2+^, Fe^3+^, respectively. The mixtures are sonicated before fluorescence measurements. Compared with the original spectra, the metal ions cause different changes in the fluorescence intensity of 1. As depicted in Fig. 4a, the fluorescence intensity of 1 remains steady for Co^2+^, NH_4_^+^, Mg^2+^ and Cr^3+^ ions; Pb^2+^, Ag^+^, Na^+^, Cd^2+^, Zn^2+^, Cu^2+^, Li^+^, Ca^2+^, K^+^, Mn^2+^ and Ni^2+^ ions produce moderate quenching; whereas Fe^3+^ ions represent manifest quenching with an efficiency of as much as 97% in contrast to that of itself.

Furthermore, competitive experiments were also performed to investigate the effect of mixed cations on emission intensity. Therefore, the sample of 1 was submerged into kinds of aqueous solutions containing Fe^3+^ ions and other metal ions (Co^2+^, NH_4_^+^, Mg^2+^, Cr^3+^, Pb^2+^, Ag^+^, Na^+^, Cd^2+^, Zn^2+^, Cu^2+^, Li^+^, Ca^2+^, K^+^, Mn^2+^ and Ni^2+^), respectively. The quenching phenomenon of Fe^3+^ ions on 1 is not affected by other competitive anions, revealing that 1 can be considered as a potential luminescent probe to detect Fe^3+^ ions among the above-mentioned cations (Fig. 4b).

**Fig. 5:**
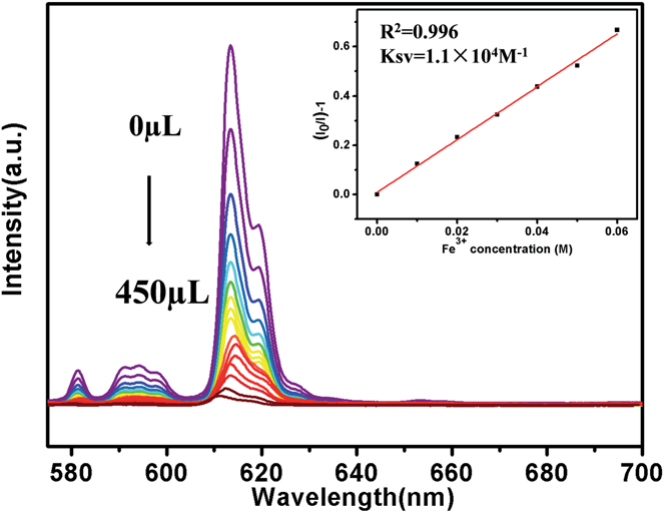
Luminescence spectra of 1 in aqueous solutions with Fe^3+^ at different concentrations (The inset is Stern–Volmer plot).

To further investigate the luminescence quenching of Fe^3+^ ions in 1, titration experiments were executed (Fig. 5). Specifically, with the increase of Fe^3+^ ions concentration, the luminescence intensity gradually decreases. Quantitatively, quenching efficiency is calculated using the Stern–Volmer (S–V) equation: (*I*_0_/*I*) = 1 + *K*_sv_[Q],^33,34^ where *I*_0_ and *I* are the luminescence intensity before and after adding metal ions, respectively, [Q] refers to the molar concentration of metal ions and *K*_sv_ is the quenching constant, which is an important indicator of the sensing ability of a fluorescent sensor. The *K*_sv_ values are found to be 1.1 × 10^4^ M^−1^ for 1, signifying that Fe^3+^ has high-efficiency quenching effect on the luminescence emission of 1. According to the ratio of 3*δ*/*K*_sv_ (*δ*: standard error),^35,36^ the detection limit of 3.5 × 10^−5^ M is superior to that of most known probes with good Fe^3+^ sensing properties (Table S3†).

**Fig. 6:**
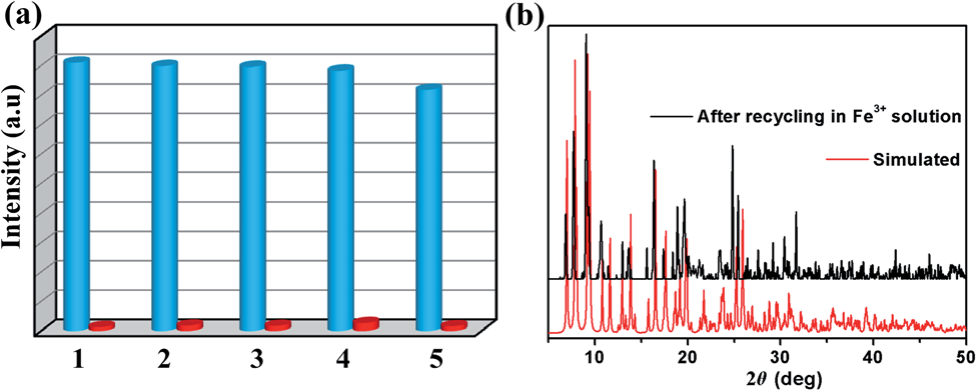
(a) Recyclability of the quenching ability of 1 immersed in water and in the presence of 0.01 M aqueous solution of Fe^3+^ ions (The blue columns represent the initial fluorescence intensity, and the red columns represent the intensity upon the addition of an aqueous solution of 0.01 M Fe^3+^ ion). (b) PXRD patterns of 1 treated by the Fe^3+^ aqueous solution.

Repeatability is an important parameter to measure the practicability of a luminescence probe.^37^ Therefore, the recyclability experiment to detect Fe^3+^ was measured as follows: samples of 1 were immersed in water for several minutes to form 1-Fe^3+^, and then 1-Fe^3+^ were washed several times and centrifuged. Subsequently, the reclaimed samples were performed by luminescence spectra. As shown in Fig. 6a, 1 can be simply and quickly regenerated and reused at five times. Meanwhile, the PXRD patterns proved that the crystallinity and structural integrity of 1 exhibited no change after the detection test (Fig. 6b), suggesting excellent recyclability.

**Fig. 7:**
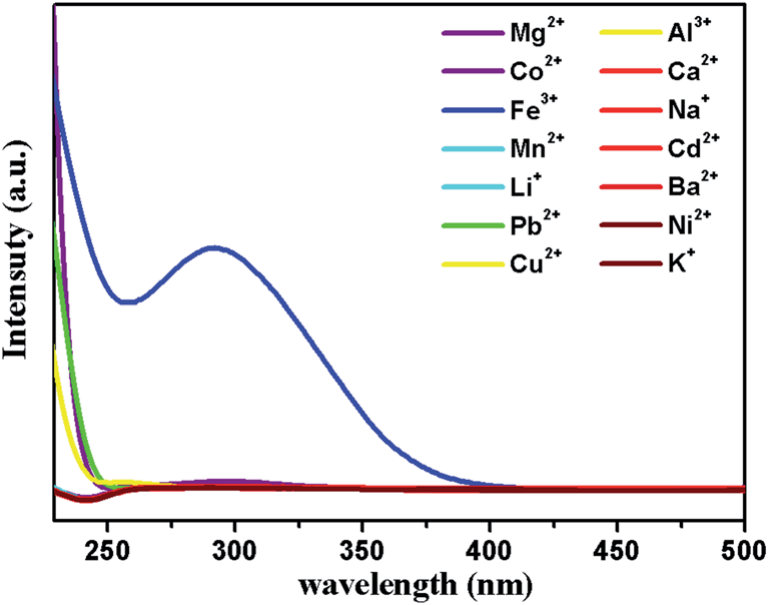
UV-vis adsorption spectra of M(NO_3_)_*x*_ aqueous solutions.

The mechanism of luminescence quenching includes several mechanisms possibilities which have been revealed by previous studies.^38–41^ To understand the underlying sensing mechanism, further analyses were carried out. First, the PXRD patterns of 1 before and after the fluorescence experiments were consistent, excluding the possibility of structural damage collapse of the framework (Fig. 6b). Second, the absorption spectrum of Fe^3+^ shows intense absorption bands in the ranges of 230–260 and 260–390 nm, and the excitation wavelength of 1 is 366 nm, which reveals that Fe^3+^ can significantly absorb the energy of the excitation wavelength, resulting in the luminescence quenching of 1 (Fig. 7). Third, the intensity ratio (*I*_0_/*I*) and Fe^3+^ concentration can be well fitted into the (*I*_0_/*I*) = 1 + *K*_sv_[Q] equation, suggesting that luminescence quenching can be put down to the dynamic process (the collision interactions). In conclusion, not only the overlap of absorption spectrum between Fe^3+^ and 1 but also the collision interactions collectively result in the luminescence quenching.

## Conclusions

In summary, a novel Eu-complex assembled from mixed ligands of BTFA and dpq have been successfully isolated and structurally characterized. The stability and luminescence properties of the synthetic Eu-complex are investigated. The complex presents a potential for highly sensitive and selective detection of Fe^3+^ in water. The quenching efficiency of Fe^3+^ is 97% which is the highest one compared to that of other metal cations. Moreover, verified by the quenching testes and titration experiments, complex 1 possesses satisfactory sensitivity and selectivity indicated by the large quenching constant of 1.1 × 10^4^ M^−1^ and prominent detection limit of 3.5 × 10^−5^ M, respectively. Most importantly, the solid products of Eu-complex can keep its original network and be recycled at least five times in the detecting experiments, which can be used as chemical sensors for practical applications. Additionally, the possible fluorescence quenching mechanism has been discussed. The successful assembly of this case may provide a good guideline to construct new Ln^III^-complexes functional materials.

## Conflicts of interest

There are no conflicts to declare.

## Supplementary Material

RA-010-D0RA03821K-s001

RA-010-D0RA03821K-s002
