# Supplementary material for: A dual-sensitized luminescent europium(iii) complex as a photoluminescent probe for selectively detecting Fe3+
Source: RSC Adv. 2020 Jun 25;10(41):24244–50. doi: 10.1039/d0ra03821k (PMC9055115; doi:10.1039/d0ra03821k)
Supplement: RA-010-D0RA03821K-s001 [file RA-010-D0RA03821K-s001.pdf]

Electronic Supplementary Material (ESI) for RSC Advances.
This journal is © The Royal Society of Chemistry 2020

Supporting Information

A dual-sensitized luminescent Europium(III) complex as photoluminescent probe for selectively detecting Fe^{3+}

Yafeng Zhao,[a#] Yanhong Xu,[a#] Bing Xu,[c] Peipei Cen,[b*] Weiming Song,[a*] Lijuan Duan[a] and Xiangyu Liu[a,d*]

[a] College of agriculture, College of Chemistry and Chemical Engineering, Ningxia University, Yinchuan 750021, China.

[b] College of Public Health and Management, Ningxia Medical University, Yinchuan 750021, China

[c] School of Chemistry and Chemical Engineering, Xi'an University of Architecture & Technology, Xi'an 710055, China

[d] State Key Laboratory of Coordination Chemistry, Nanjing University, Nanjing, 210023, China

# These authors contributed equally to this work.

***Corresponding author**

Dr. Xiangyu Liu

Tel.: +86-951-2062004

Fax: +86-951-2062860

E-mail: xiangyuliu432@126.com

***Corresponding author**

Prof. Weiming Song

E-mail: songwm@nxu.edu.cn

***Corresponding author**

Dr. Peipei Cen

E-mail: 13895400691@163.com

Contents

Fig. S1 Molecular stacking charts of **1**.

Fig. S2 PXRD patterns of **1**.

Fig. S3 TG curve for **1**.

Fig. S4 PXRD patterns of **1** after being soaked in water.

Fig. S5 NMR spectrum of **1** after dissolving in MeOH.

Table S1. Selected bond lengths (Å) and bond angles (°) for **1**.

Table S2. The calculated results for Eu^{III} ions configuration of **1** by SHAPE 2.1 software.

Table S3. Comparison of detection capacity of **1** towards Fe^{3+} ion with other materials.

References

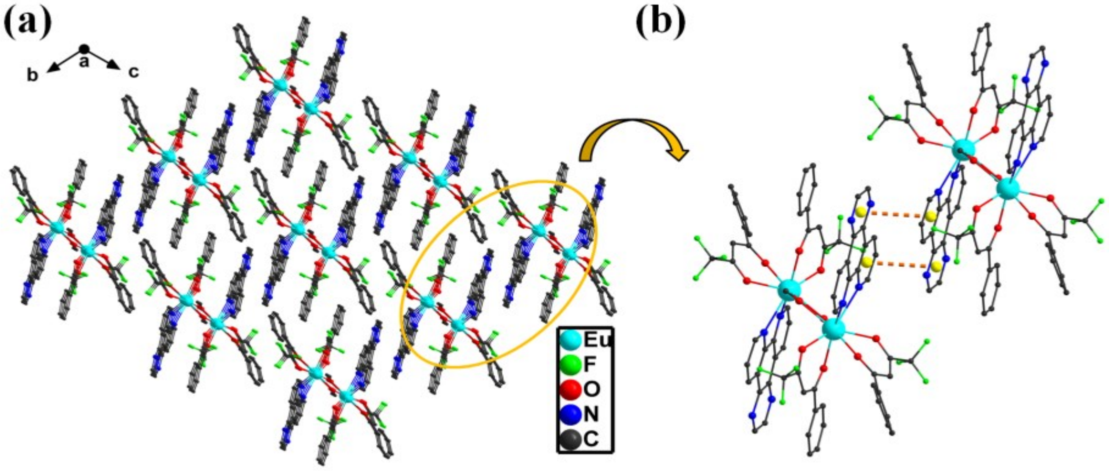

Fig. S1 Molecular stacking charts of **1**.

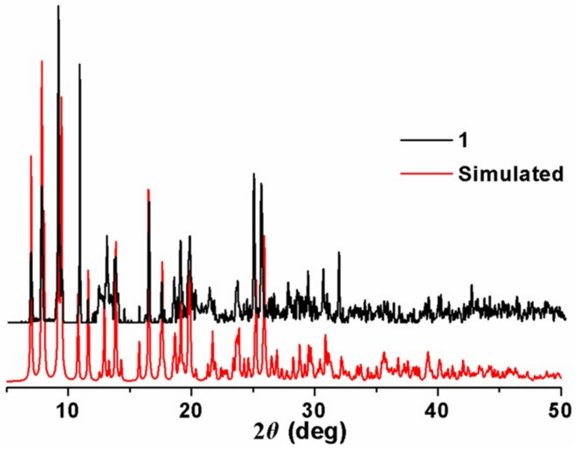

Fig. S2 PXRD patterns of **1**.

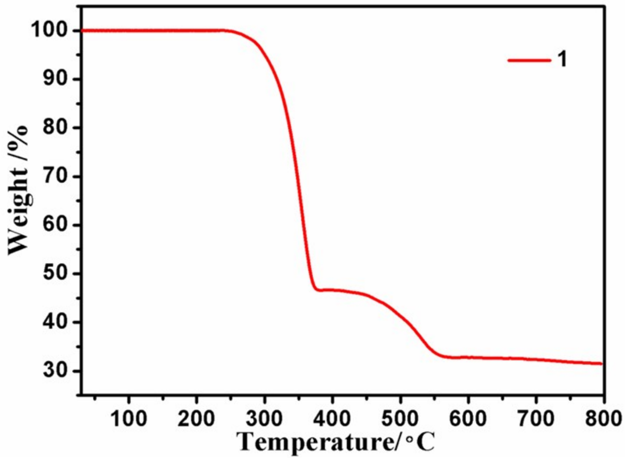

Fig. S3 TG curve for **1**.

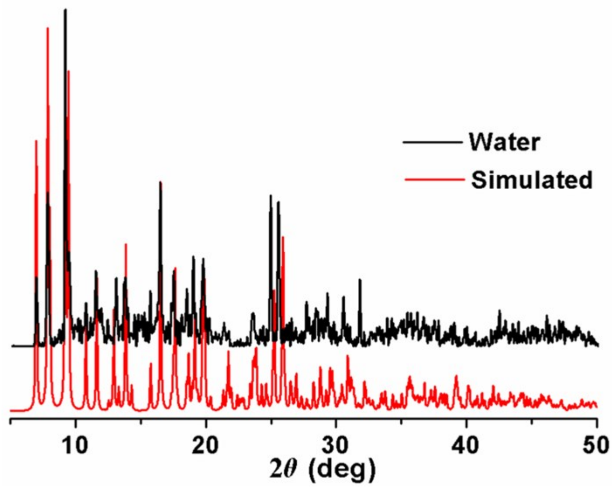

Fig. S4 PXRD patterns of **1** after being soaked in water.

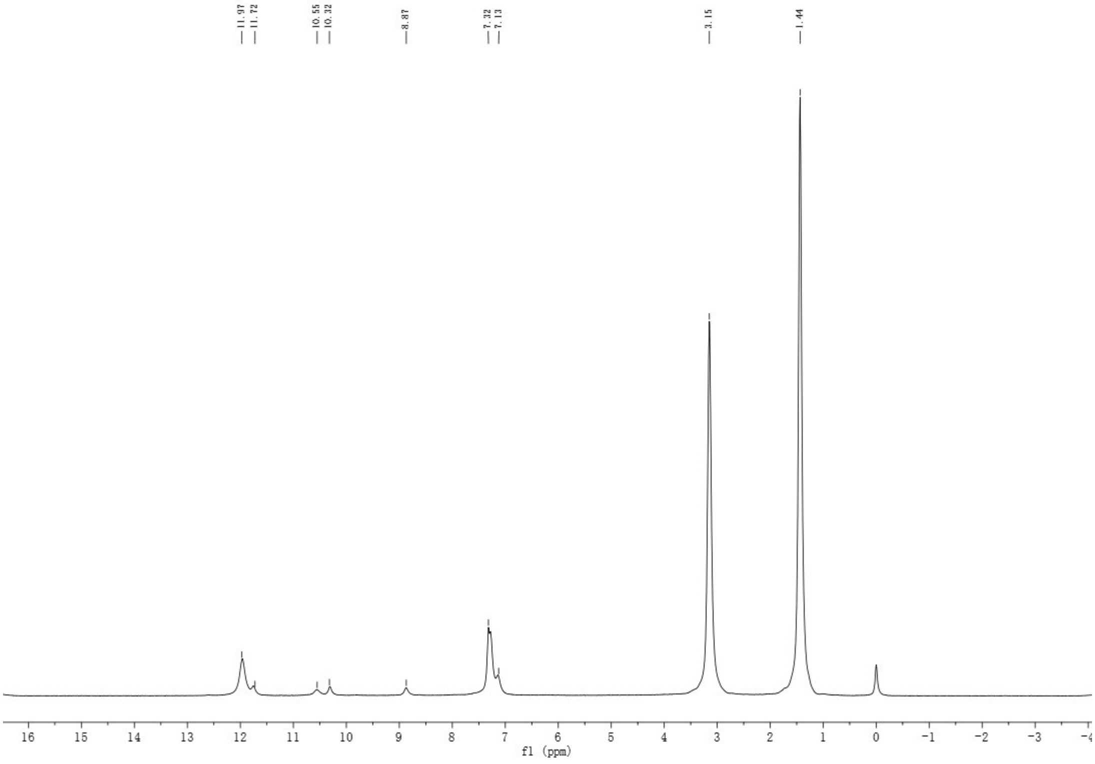

Fig. S5 NMR spectrum of **1** after dissolving in MeOH.

Table S1. Selected bond lengths (Å) and bond angles (°) for **1**.

Compound **1**			
Eu(1)-O(1)	2.385(5)	Eu(1)-O(2)	2.373(5)
Eu(1)-O(3)	2.393(5)	Eu(1)-O(4)	2.437(5)
Eu(1)-O(5)	2.312(5)	Eu (1)-O(5)#1	2.313(4)
Eu(1)-N(1)	2.624(6)	Eu(1)-N(2)	2.609(6)
O(1)-Eu(1)-O(3)	137.29(17)	O(1)-Eu(1)-O(4)	71.98(16)
O(1)-Eu(1)-N(1)	82.05(16)	O(1)-Eu(1)-N(2)	140.49(18)
O(2)-Eu(1)-O(3)	72.27(17)	O(2)-Eu(1)-O(3)	80.94(16)
O(2)-Eu(1)-O(4)	79.92(17)	O(2)-Eu(1)-N(1)	147.41(18)
O(2)-Eu(1)-N(2)	147.08(18)	O(3)-Eu(1)-O(4)	71.00(16)
O(3)-Eu(1)-N(1)	106.56(16)	O(3)-Eu(1)-N(2)	73.24(18)
O(4)-Eu(1)-N(1)	73.17(18)	O(4)-Eu(1)-N(2)	109.57(17)
O(5)-Eu(1)-O(1)	78.38(16)	O(5) #1-Eu(1)-O(1)	121.73(15)
O(5)-Eu(1)-O(2)	115.03(16)	O(5) #1-Eu(1)-O(2)	77.82(17)
O(5)-Eu(1)-O(3)	144.10(17)	O(5) #1-Eu(1)-O(3)	82.63(16)
O(5)-Eu(1)-O(4)	140.70(16)	O(5) #1-Eu(1)-O(4)	147.74(18)
O(5) -Eu(1)-O(5) #1	70.80(19)	O(5)-Eu(1)-N(1)	77.74(16)
O(5) #1-Eu(1)-N(1)	133.99(18)	O(5)-Eu(1)-N(2)	78.18(17)
O(5) #1-Eu(1)-N(2)	78.91(17)	N(2)-Eu(1)-N(1)	62.14(17)
#1 1-X,1-Y,1-Z			

Table S2. The calculated results for Eu^{III} ions configuration of **1** by SHAPE 2.1 software.

Eu^{III} ion geometry analysis of **1**.

```
HBPY-8        3 D6h   Hexagonal bipyramid
CU-8          4 Oh    Cube
SAPR-8        5 D4d   Square antiprism
TDD-8         6 D2d   Triangular dodecahedron
JGBF-8        7 D2d   Johnson gyrobifastigium J26
JETBPY-8      8 D3h   Johnson elongated triangular bipyramid J14
JBTPR-8       9 C2v   Biaugmented trigonal prism J50
BTPR-8       10 C2v   Biaugmented trigonal prism
JSD-8        11 D2d   Snub diphenoid J84
TT-8         12 Td    Triakis tetrahedron
ETBPY-8      13 D3h   Elongated trigonal bipyramid

Structure [ML8 ]   HBPY-8    CU-8     SAPR-8   TDD-8    JGBF-8    JETBPY-8   JBTPR-8   BTPR-8   JSD-8    TT-8     ETBPY-8
 ABOXIY      ,     15.765,   9.160,   0.626,   2.420,   16.045,   28.065,    2.983,    2.489,   5.294,   9.881,   23.701
```

Configuration	ABOXIY, **1**
Hexagonal bipyramid (D_{6h})	15.765
Cube (O_h)	9.160
Square antiprism (D_{4d})	**0.626**
Triangular dodecahedron (D_{2d})	2.420
Johnson gyrobifastigium J26 (D_{2d})	16.045
Johnson elongated triangular bipyramid J14 (D_{3h})	28.065
Biaugmented trigonal prism J50 (C_{2v})	2.983
Biaugmented trigonal prism (C_{2v})	2.489
Snub siphenoid J84 (D_{2d})	5.294
Triakis tetrahedron(T_d)	9.881
Elongated trigonal bipyramid(D_{3h})	23.701

Table S3. Comparison of detection capacity of **1** towards Fe^{3+} ion with other materials.

Materials	Solvent	Detection Limit	Ref.
$[Tb(tftba)_{1.5}(phen)(H_2O)]_n$	Water	1.27×10^{-5} M	1
Eu-MOF	Water	1.5×10^{-5} M	2
$[Tb(HL)(H_2O)_2(NO_3)]\cdot NO_3$	Water	1.6×10^{-5} M	3
$[Tb(HL)_{1.5}(H_2O)(DMF)]\cdot 2H_2O$	aqueous	2.0×10^{-5} M	4
$[Eu_2(BTFA)_4(OMe)_2(dpq)_2]$	Water	3.5×10^{-5} M	This work
Eu^{3+}@MIL-53-COOH (Al)	Water	5.0×10^{-5} M	5
BUT-15	Water	8.0×10^{-5} M	6
$[Eu(bpda)_{1.5}]\cdot H_2O_n$	Water	9.0×10^{-5} M	7
$\{[Cd_3(HL)_2(H_2O)_3]\cdot 3H_2O\cdot 2CH_3CN\}_n$	Water	9.06×10^{-5} M	8
$[Zn_5(hfipbb)_4(trz)_2(H_2O)_2]_n$	Water	2.0×10^{-4} M	9
$\{[Cd(5\text{-asba})(bimbu)]\}_n$	Water	1.87×10^{-4} M	10
EuL_3	Ethanol	10^{-4} M	11
$Eu(acac)_3{\subset}Zn(C_{15}H_{12}NO_2)_2$	DMF	5.0×10^{-3} M	12

References:

(1) H.-H. Yu, J.-Q. Chi, Z.-M. Su, X. Li, J. Sun, C. Zhou and Q. Liu, *CrystEngComm.*, 2020, 3638-3643.

(2) W. Yang, J. Li, Z. Xu, J. Yang, Y. Liu and L. Liu, *J. Mater. Chem. C.*, 2019, **7**, 10297-10308.

(3) W. Gao, F. Liu, B.-Y. Zhang, X.-M. Zhang, J.-P. Liu, E.-Q. Gao and Q.-Y. Gao, *Dalton Trans.*, 2017, **46**, 13878-13887.

(4) F. Zhao, X.-Y. Guo, Z.-P. Dong, Z.-L. Liu and Y.-Q. Wang, *Dalton Trans.*, 2020, **47**, 8972-8982.

(5)Y. Zhou, H.-H. Chen and B. Yan, *J. Mater. Chem. A.,* 2014*,* **2***,* 13691-13697.

(6) H.-J. Zhang, R.-Q. Fan, W. Chen, J.-Z. Fan, Y. W.-Dong, Y. Song, X.-Du, P. Wang and Y. L. Yang, *Cryst. Growth Des.,* 2016, **16**, 5429-5440.

(7) J. Wang, J.-R. Wang, Y. Li, M. Jiang, L.-W. Zhang and P.-Y. Wu, *New J. Chem.,* 2016, **40**,

8600-8606.

(8) W. Q. Tong, W.-N. Liu, J. G. Cheng, P.-F. Zhang, G.-P. Li, L. Hou and Y.-Y. Wang, *Dalton Trans.*, 2018, **47**, 9466-9473.

(9) B.-L. Hou, D. Tian, J. Liu, L.-Z. Dong, S.-L. Li, D.-S. Li and Y.-Q. Lan, *Inorg. Chem.*, 2016, **55**, 10580-10586.

(10) Y.-J. Yang, M.-J. Wang and K.-L. Zhang, *J. Mater. Chem. C*, 2016, **4**, 11404-11418.

(11) M. Zheng, H. Tan, Z. Xie, L. Zhang, X. Jing and Z. Sun, *ACS Appl. Mater. Interfaces*., 2013, **5**, 1078-1083.

(12) Q. Tang, S. Liu, Y. Liu, J. Miao, S. Li, L. Zhang, Z. Shi and Z. Zheng, *Inorg. Chem.*, 2013, **52**, 2799-2801.
